# The fluctuating ribosome: thermal molecular dynamics characterized by neutron scattering

**DOI:** 10.1038/srep37138

**Published:** 2016-11-16

**Authors:** Giuseppe Zaccai, Francesca Natali, Judith Peters, Martina Řihová, Ella Zimmerman, J. Ollivier, J. Combet, Marie-Christine Maurel, Anat Bashan, Ada Yonath

**Affiliations:** 1 Institut Laue Langevin, F-38042 Grenoble, France; 2 Institut de Biologie Structurale (IBS), Univ. Grenoble Alpes, CEA, CNRS, 38044 Grenoble, France; 3 CNR-IOM, OGG, F-38042 Grenoble, France; 4 Univ. Grenoble Alpes, LiPhy, F-38044 Grenoble, France; 5 Institut de Systématique, Evolution, Biodiversité, ISYEB - UMR 7205- CNRS, MNHN, UPMC, EPHE UPMC, Sorbonne Universités, 57 rue Cuvier, CP 50, 75005 Paris, France; 6 Institute of Physics, Charles University, Faculty of Mathematics and Physics, CZ-121 16 Prague, Czech Republic; 7 Weizmann Institute, Department of Structural Biology, 76100 Rehovot, Israel; 8 Institut Charles Sadron, CNRS-UdS, 67034 Strasbourg Cedex 2, France

## Abstract

Conformational changes associated with ribosome function have been identified by X-ray crystallography and cryo-electron microscopy. These methods, however, inform poorly on timescales. Neutron scattering is well adapted for direct measurements of thermal molecular dynamics, the ‘lubricant’ for the conformational fluctuations required for biological activity. The method was applied to compare water dynamics and conformational fluctuations in the 30 S and 50 S ribosomal subunits from *Haloarcula marismortui*, under high salt, stable conditions. Similar free and hydration water diffusion parameters are found for both subunits. With respect to the 50 S subunit, the 30 S is characterized by a softer force constant and larger mean square displacements (MSD), which would facilitate conformational adjustments required for messenger and transfer RNA binding. It has been shown previously that systems from mesophiles and extremophiles are adapted to have similar MSD under their respective physiological conditions. This suggests that the results presented are not specific to halophiles in high salt but a general property of ribosome dynamics under corresponding, active conditions. The current study opens new perspectives for neutron scattering characterization of component functional molecular dynamics within the ribosome.

Ribosomes are universal cellular complexes made up of many different ribosomal proteins and long ribosomal ribonucleic acid (RNA) chains that enable decoding, successive peptide bond formation and the protection of nascent protein chains. They are present as a two-subunit structure in all living organisms with adaptive characteristics that make them fit to each physiological environment. Ribosomal proteins stabilize the ribosome structure or assist its activity, yet none plays a direct role in peptide bond formation or polypeptide elongation. The ribosome can be considered, therefore, as a giant ribozyme complex in which RNA controls catalytic activity^1^,^2^. The structural bases of mechanisms involved in ribosome function are studied intensively and observations of various conformations by cryo-electron microscopy (cryo-EM) and high-resolution X-ray crystallography established that during its functional cycle the ribosome undergoes conformational changes, ranging from local group reorientation to large domain movements^3^,^4^,^5^,^6^,^7^. During ribosome crystallization trials, metal clusters used to provide anomalous phasing power were found to extend dramatically the resolution of the diffraction, through a reduction of subunit internal flexibility, “by ‘gluing’ symmetrical related subunits”^3^. It has, furthermore, been shown that ribosome complexes with mRNA and tRNA gave better resolution than apo ribosome structures in X-ray crystallographic structures, suggesting that substrate binding helps stabilize subunits in a preferred conformation to enable more homogeneous samples for crystallization^8^,^9^. Comparisons between ribosomal subunit structures from mesophile and thermophile bacteria, as well as archaeal halophiles indicated that inherently flexible elements are involved in inter-subunit contacts and substrate binding^10^,^11^, leading Horan *et al*.^10^ to conclude that ‘inter-subunit movement is required for ribosome translocation, accounting for the universal two-subunit architecture of ribosomes’. In addition to the extensive structure-function studies, a variety of solvent conditions has been characterized and associated with halophile ribosomal subunit structural and functional integrity, during the extraordinary scientific effort that led to their crystallization^11^. All these data show that a detailed description of the ribosome requires an understanding in terms of dynamics.

Dynamics refers to the forces that stabilize structure and define motions. On one hand, single particle cryo-EM and X-ray structural studies, while informing on the conformational change amplitudes, do not contain information on forces or time-scales. On the other hand, molecular dynamics (MD) simulations and normal mode calculations are particularly challenging because of the size and complexity of ribosome structures^12^,^13^,^14^,^15^,^16^,^17^. Dynamics is also very sensitive to environment, which leads to a well-structured system being inactive under certain solvent conditions^18^, and it is now fully accepted that further to structure being adapted to function, appropriate molecular motions are also necessary. Molecular dynamics on the picosecond to nanosecond time scale acts as the ‘lubricant’ of functional conformational changes on longer time scales^19^,^20^,^21^. Following neutron scattering experiments, it has been proposed that adaptation occurs *via* evolutionary selection of dynamics on the thermal timescale^22^.

Neutron beams provide a soft radiation of wavelength and energy similar in magnitude to molecular thermal fluctuation amplitudes and energies, respectively, which inflicts practically no radiation damage on a biological sample. In a neutron scattering experiment, mean square displacements (MSD) and the effective force constant anchoring atoms in a structure are calculated from measured energy and momentum changes of the neutron as a function of temperature. To use a mechanical analogy: in a collision between a moving billiard ball and one that is anchored by a spring, the force constant of the spring as well as the vibration amplitude of the anchored ball can be calculated from the changes in energy and momentum of the moving ball after it is ‘scattered’. Systems studied by neutron scattering include intrinsically disordered proteins, whole cells, membranes, nanoparticles and tissue^22^,^23^,^24^,^25^,^26^,^27^.

The current study presents neutron scattering results that enabled the determination of global thermal dynamics in halophilic ribosomal subunit samples^28^. Halophiles were chosen because of sample stability. They also respond to solvent effects^29^,^30^, which will be applied in future experiments to explore dynamics/structure/function relations in more detail. Previous neutron scattering experiments on live cells with different physiological temperatures, demonstrated adaption of MD forces to yield similar mean square displacements (MSD) under active conditions^22^; *e.g.* psychrophiles at low temperature are ‘softer’ in order to have similar MSD to hyperthermophiles at high temperature. We would expect, therefore, that the results on halophiles would be more generally applicable than just reflecting an extremophile property, provided the measurements were performed in high salt active conditions.

H30S (*Haloarcula marismortui* 30 S subunit) and H50S (*Haloarcula marismortui* 50S subunit) dynamics was measured on the complementary ~10 picosecond and nanosecond timescales, corresponding, respectively, to water diffusion and fast local vibrations^20^,^31^, on the one hand, and side-chain conformational sampling, on the other. Free and hydration water are found with similar motion parameters in both H30S and H50S. A marginal solvent effect explored on H30S samples suggested proportionally more bound water in 3 M NaCl compared to 3 M KCl, correlated with stronger hydration interactions of the Na^+^ ion, as observed previously in halophilic proteins^30^. On the nanosecond timescale, effective force constants (resilience) from the temperature dependence of the fluctuations were calculated from the temperature dependence of the fluctuations. H30S is revealed to have softer resilience and larger mean square fluctuations than H50S. The results are discussed in terms of the role of dynamics in RNA activity and of the differential functional flexibility of 30 S *versus* 50 S subunits, for example, to facilitate conformational adjustments required for messenger and transfer RNA binding. The measured dynamics in the different timescales can, furthermore, serve as quantitative experimental input for coarse-grained MD simulations, to provide further understanding of the ribosome as a vital molecular machine.

## Results

### Thermal vibrations on the ~10 picosecond timescale are similar for both subunits

**Figure 1:**
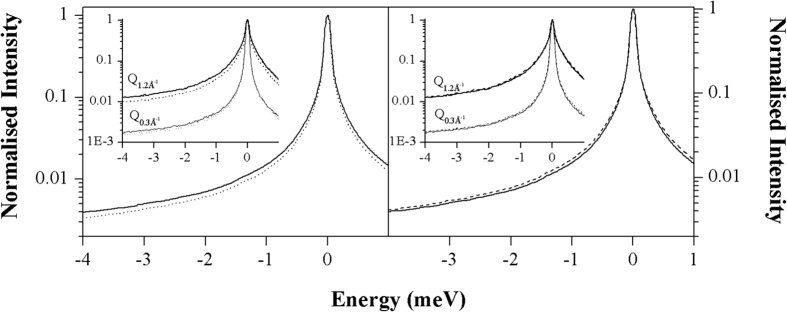
Quasi-elastic neutron scattering intensities, at 298 K, binned over the whole wave-vector range accessible on the IN5 spectrometer (0.2–2 Å^−1^) and normalized to unity. Left panel: H30S-3 M NaCl (solid line) and H50S-3 M NaCl (dotted line), Right panel: – H30S-3 M NaCl (solid line) and H30S-3 M KCl (dashed line). Insets: same spectra at reference Q = 0.3 Å^−1^ (fluctuation amplitudes > 20 Å) and *Q* = 1.2 Å^−1^ (amplitudes ~5 Å). A broader intensity distribution indicates scattering from faster motions (see text).

Quasi-elastic neutron scattering (QENS) spectra measured on the timescale of fast thermal dynamics are given in Fig. 1.

The left panel shows the comparison H30s *versus* H50S, both in 3 M NaCl in H_2_O. Visual inspection reveals a slightly broader spectrum for H30S-3 M NaCl *vs* H50S-3M NaCl, indicating marginally faster average dynamics in the 30S subunit. All samples were equilibrated in H_2_O, avoiding the use of D_2_O (see Methods). In H_2_O containing samples on the 10 ps timescale – a few Å length scale, the scattering signal is dominated by the diffusion of free and hydration H_2_O in the samples and fast motions within the ribosomal subunits (see ‘Theoretical background’ in Methods). Water content in the samples was 65% for H30S-3 M NaCl, and 67% for H50S-3 M NaCl (see Methods), so that the apparently ‘faster’ 30S subunit cannot be accounted for by the total water content in the sample, since it is 2% lower than for the 50 S, and is likely due to faster motions of groups within the subunit.

In further analysis given in Supplementary Information (SI), the QENS was fitted to yield two diffusive populations with parameters given in Table S1. The model used for the analysis is described in detail in SI. From the values of the diffusion coefficients, the populations are likely to correspond to free and hydration water, respectively, as previously published by Bellissent-Funel and collaborators^32^, with motion parameters that lie within errors for H30S and H50S. Analysis of the elastic intensity yields the proportion of H atoms seen as ‘immobile’ (*i.e.* moving too slowly to be resolved in the experiment, see SI). These were 6.3 ± 0.5% for H30S and 9.3 ± 0.3% for H50S, indicating a larger proportion of atoms in H50S compared to H30S that are from domains in which atomic motions are too slow to be resolved on the ~10 ps time scale. The faster average motions in 30 S could, therefore, be a reflection of fewer ‘immobile’ atoms on this timescale compared to the 50 S. Considering also that the ‘faster’ 30 S contains 2% less total water, a complementary interpretation may be that the ratio free-to-bound water in the 30S is higher than in the 50S (*i.e.* the 50S binds more water).

A small solvent effect is observed in Fig. 1 (right panel), where a marginally broader spectrum (faster dynamics) is observed for H30S-3 M KCl sample compared to H30S-3 M NaCl. The role of high K^+^ ion intracellular concentrations in halophiles has been discussed previously in terms of ion binding and hydration^33^,^34^. Water content in the H30S-3 M KCl and H30S-3 M NaCl was 62% and 65%, respectively (see Methods). Here again, the effect is small and the sample with less water appears to have faster dynamics. We tentatively propose an interpretation in terms of the different water binding properties of K^+^ and Na^+^ ions, respectively. Because of the stronger hydration interactions of Na^+^ compared to K^+^, we would expect a relatively higher ratio of free to bound water in the KCl sample, which would account for the ‘faster’ dynamics. Stronger hydration interactions in NaCl compared to KCl have been observed in a previous neutron scattering dynamics study of malate dehydrogenase from *Haloarcula marismortui*^35^. And different hydration effects between tRNA solutions in NaCl and KCl solvents have been reported from a small angle neutron scattering study^36^.

### H30S is significantly softer with larger MSD than H50S (both in 3 M salt) on the nanosecond timescale of conformational sampling

**Figure 2:**
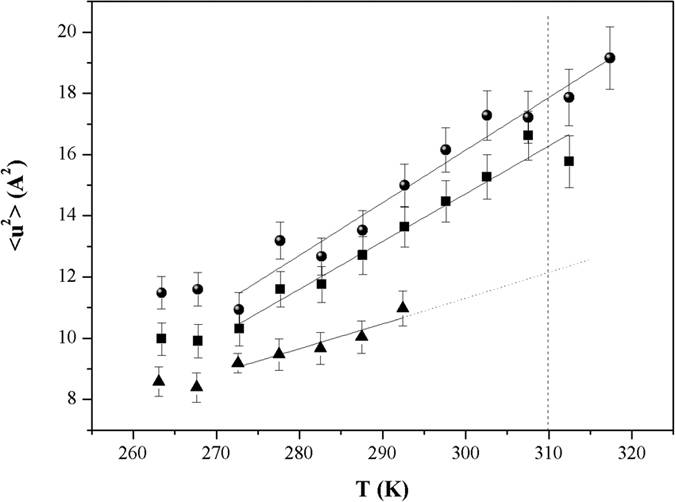
Mean square displacements (MSD) on the nanosecond timescale of H30S-3 M NaCl (circles), H30S-3 M KCl (squares) and H50S-3 M NaCl (triangles) extracted from elastic intensities in the wave-vector range 0.34–0.85 A^−1^. The solid lines represent the best fits for data above 270 K (see text), while the dashed line corresponds to the MSD at the reference value of 37 °C. A steeper slope with temperature indicates a softer effective force constant for the motions (see text).

Temperature dependence elastic scans on H30S and H50S were recorded on the IN16 spectrometer at ILL (see Methods). MSD *versus* temperature plots for the three samples are shown in Fig. 2. The observed dynamics is dominated by chemical group motions within the ribosomal subunits because free H_2_O and hydration water diffusion, observed by QENS on IN5, lie outside the elastic window ~1 ns - ~5 Å time- length-scale of the IN16 spectrometer^32^,^37^. Very slow water molecules that are strongly bound to ionic groups within the ribosomal subunits are considered as part of the biological complex. All samples in Fig. 2 indicate a flatter MSD below ~273 K, which may be due to a ‘stiffening’ of the ribosome subunit complexes due to freezing of free water in the samples. We, therefore, performed straight-line fits to the MSD beyond the kink at ~273 K to provide absolute scale values for the effective force constants (resilience, 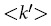 in N/m) in this timescale (see Methods). The 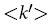 values calculated from the MSD temperature dependence are in Table 1. NaCl and KCl sample values are within errors, indicating there is no solvent effect in this timescale except for slightly lower MSD for the KCl condition, which may be due to lower macromolecular plasticity in the drier sample (62% hydration compared to 65% for the sample in NaCl, see Methods). The effective force constant (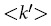) is a factor of two smaller (softer) and the MSD at 37 °C significantly higher for H30S compared to the H50S both in 3 M NaCl. This difference cannot be due to water content because the ‘softer’ 30 S sample is drier (65% water content) than the 50 S (67%).

Compared to previous studies on the same timescale for a membrane^38^ and an intrinsically disordered protein^39^, the MSD values for the ribosomal subunits are significantly higher and the resilience values significantly lower. This is certainly due to the higher H_2_O content in the ribosome samples allowing the observation of large amplitude, softer diffusive motions. Indeed, an IN16 study of HDL (high density lipoprotein) in solution yielded MSD and resilience values of the same order as calculated from the ribosome data (J. Peters, private communication).

## Discussion

In their 2015 review, Herschlag *et al*.^40^ write that “To understand RNA, it is necessary to move beyond a descriptive categorization towards quantitative predictions of its molecular conformations and functional behavior.” The plasticity of RNA structure has been demonstrated in the first decade after the first crystallographic structure of tRNA, including by small angle neutron scattering experiments^41^. Ribozyme motifs constitute evolved RNA molecules that carry out identical chemical functionality and represent ideal systems to study underlying structure-function relationships, illustrating the diversity of RNA’s functional role in biology (review by Fürtig *et al*.^42^). In a time-resolved NMR spectroscopy study of the adenine-dependent hairpin ribozyme, it has been shown that high activation barriers have to be overcome to populate active states with short life times^43^ and concluded that conformational dynamics, not chemistry, constitute the catalytic rate-limiting step. Fischer *et al*.^6^ have followed tRNA movement through the ribosome during translocation by time-resolved single-particle cryo-EM, and images of intermediate states revealed dynamic interactions between tRNA and ribosomal residues in a flat free energy landscape at physiological temperature. The authors concluded that “The ribosome functions as a Brownian machine that couples spontaneous conformational changes driven by thermal energy to directed movement”. The results from the various studies triggered further studies on the molecular dynamics of ribosomes in terms of forces and thermal energies by MD simulations (see references in the Introduction) and the present neutron scattering study, which delivers quantitative absolute scale values that could be used as input and/or experimental checks in computational studies of the ribosome, as was done, for example, in a previous study by Stadler *et al*.^44^ comparing dynamics of hemoglobin from different species, in which force constants measured by neutron scattering guided a coarse-grained elastic network approach to provide an understanding of the functional dynamics differences at the amino acid level^44^.

QENS results from IN5 and MSD results IN16 are complementary, informing on different dynamics. On the ~10 ps timescale, QENS is dominated by free and hydration water diffusion in the samples. Similar motion parameters were found for H30S and H50S, in 3 M NaCl. A comparison of H30S in 3 M NaCl and 3 M KCl suggested a higher ratio of free to bound water in the KCl sample in accordance with the stronger hydration interactions of the Na^+^ ion. Analysis of the elastic component on the 10ps timescale indicated a higher proportion (~9% *cf* ~6%) of atoms that appear to be immobile. This relative ‘immobility’ of the 50S in the fast IN5 timescale is reflected in the longer timescale of IN16, sampling large amplitude side chain motions, in which the mean effective force constant for nanosecond motions in H50S is a factor of 2 ‘stiffer’ than for H30S, while mean square displacements are a factor of 1.5 larger for H30S. These are significant differences indicating a flatter energy landscape for the small subunit that may play a functional role in initial mRNA and tRNA binding. The observations are likely be related to the larger amino acid/nucleotide ratio and solvent interaction area in H30S. H atoms in RNA groups have been shown in MD simulations as well as by neutron scattering to have stiffer dynamics^45^. The observation that H30S is softer than H50S is, therefore, in accordance with its higher nucleotide/(amino acid residue) ratio, 0.60 for 50S compared to 0.46 for 30 S. The larger surface to volume ratio in H30S compared to H50S would also contribute to a softer structure through a larger area of solvent interactions. They reveal the dynamic nature of conformational flexibility observed for the small subunit by crystallization trials, X-ray crystallography and cryo-EM. In the hypothesis that dynamics is similarly adapted to function under physiological conditions of temperature and solvent^11^,^22^, we expect the results obtained on the halophile ribosomal subunits in high salt to be generally applicable; *i.e.* 30S subunits are softer than 50 S for mesophiles under mesophile conditions, for thermophiles under thermophile conditions *etc.* The work could, therefore, pave the way for the design of ribosome inhibitors that would act by stiffening small subunit dynamics.

The current study opens up important perspectives for the experimental characterization of ribosome functional molecular dynamics. Since the scattering cross-section of deuterium is much weaker than that of natural abundance hydrogen, neutron scattering using specific deuterium labeling is a powerful method to study motions in parts of a complex system. This was done, for example, to measure the motions in the active core of the membrane protein bacteriorhodopsin^46^. Deuterium labelling of ribosomes has a long history^47^ and can be applied to observe separately the dynamics of different ribosome components (RNA, protein, bound tRNA…), with the aim of better characterising the dynamics interactions in a working ribosome as well as to relate the experimental observations with coarse-grained MD simulations, currently being developed by others.

## Materials and Methods

### Sample preparation

Ribosomes and ribosomal subunits from *Haloarcula marismortui* were obtained as described in Harms *et al*.^28^. Neutron scattering experiments can profit from the different scattering cross-sections of natural abundance hydrogen and deuterium in order to reduce or enhance the contribution of water to the signal. It is now well accepted, however, that the essential role of H-bonds in protein stabilization and dynamics can lead to significantly different dynamics in H_2_O and D_2_O as shown in previous thermodynamics and neutron scattering studies^29^,^35^. We were anxious to stay as close to dynamics under physiological conditions as possible so we avoided D_2_O, and included a slight excess of H_2_O in the samples. About 100 mg of each sample in either 3 M NaCl or 3 M KCl solvent, in which halophilic ribosomes are structurally stable, was equilibrated in H_2_O atmosphere to the following hydration levels: 65% for H30S-3 M NaCl, 62% for H30S-3 M KCl and 67% for H50S-3 M NaCl. Samples were sealed in vacuum-tight flat aluminum holders. In order to verify that no loss of material had occurred and that the hydration state was maintained, samples were weighed before and after the neutron scattering experiments.

### Neutron scattering experiments

Quasi elastic neutron scattering (QENS) spectra were collected on the high-resolution time–of–flight spectrometer IN5^48^ at the ILL in Grenoble. IN5 is a disk chopper time-of-flight spectrometer, which spans a wide range of incident wavelengths and energy resolutions. Neutrons, scattered at the sample, are detected at 4 m distance by 259 spherically ordered ^3^He detectors of the height of 3 m, covering an angular range between 14.5° and 132.5°. In addition, a position sensitive detector (PSD) counts neutrons in the small angle region between 2° and 8°. The instrument was used at two incident wavelengths of 5.1 and 10 Å providing the energy resolution of 75 and 12 μeV (FWHM) and the momentum transfer range 0.21 < Q < 1.98 Å^–1^ and 0.1 < Q < 1.01 Å^–1^, respectively.

Elastic (zero energy transfer, within instrumental resolution) spectra as a function of temperature were acquired on the cold backscattering spectrometer IN16^49^ at ILL, with an incident wavelength of λ = 6.27 Å and energy resolution of 0.9 μeV (FWHM) corresponding to a time window of about 1 ns and a momentum transfer (*Q*) range from 0.19 to 1.89 Å^−1^.

Data correction on both IN5 and IN16 was performed using LAMP^50^ to correct for transmission, normalize the raw data to the neutron flux, subtract the background due to the empty cell, correct for detector efficiency and energy resolution by using vanadium, a purely elastic, incoherent scatterer with a constant signal as a function of (*Q*)^37^. Transmission values were all above 0.9 so that multiple scattering effects were not taken into consideration for the data treatment.

### Theoretical background

Neutron scattering data are sensitive mainly to hydrogen motions, because the incoherent neutron scattering cross-section of ^1^H is an order of magnitude larger than that of other nuclei in biological samples. Hydrogen atoms are homogeneously distributed in biological macromolecules, however, and are representative of global averaged molecular dynamics^51^. H-bond diffusive dynamics is mainly observed on the picosecond timescale, while, on longer timescales, hydrogen atoms reflect the conformational sampling motions of the groups to which they are bound^51^.

The scattered elastic incoherent intensity can be described in a Gaussian approximation by the dynamic structure factor at zero energy exchange^52^,^53^


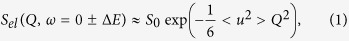


where *ΔE* is instrumental energy resolution, related to the time window through Heisenberg’s uncertainty principle, and <*u*^2^> is the average time-dependent atomic mean square displacement (MSD)^54^,^55^ in the limit defined by *ΔE*, the energy resolution of the spectrometer. (Note that with respect to the radius of gyration of the motion about its center of mass (*R*_g_), <*u*^2^> = 2*R*_g_^2^).

Similarly to the Guinier approximation in small angle scattering, the *Q* range of validity for the Gaussian approximation depends on the geometry of the motion and could go as far as <*u*^2^> *Q*^2^ ≈ 4^56^. The <*u*^2^> can be obtained for each temperature by the slope of the semi-logarithmic plot of the incoherent scattering function through


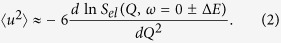


Note that in studies where temperature scan elastic window data are collected down to 20 K the absolute scale calibration is performed through normalization to the scattering curve at 20 K (*e.g.* see Wood *et al*.^57^). At this temperature the MSD is essentially zero leading to a theoretically constant ln *S*_*el*_ *vs Q*^2^. In the current study, it was important to remain above freezing temperature to avoid sample damage. At the lowest measured temperature, the MSD value is ~10 Å, very far from zero. The ln *S*_*el*_ *vs Q*^2^ slope values are, therefore, calibrated to an absolute Å^2^ scale through the division by the elastic incoherent vanadium scattering that is constant as a function of (*Q*) (see ‘Neutron scattering experiments’, above).

An effective average force constant for sample dynamics, <*k*>, can be calculated from the slope of <*u*^2^> as a function of temperature, by applying a quasi-harmonic approximation^58^:


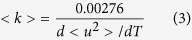


In equation (3), the numerical constant is for <*k*> expressed in Newton per meter when <*u*^2^> is given in Ångstrom squared and *T* is the temperature in Kelvin.

## Additional Information

**How to cite this article**: Zaccai, G. *et al*. The fluctuating ribosome: thermal molecular dynamics characterized by neutron scattering. *Sci. Rep.* **6**, 37138; doi: 10.1038/srep37138 (2016).

**Publisher’s note**: Springer Nature remains neutral with regard to jurisdictional claims in published maps and institutional affiliations.

## Supplementary Material

Supplementary Information

## Figures and Tables

**Table 1:** <*k*> values calculated from the MSD temperature dependence (equation 3) and MSD at reference temperature value of 37 °C, in the nanosecond time scale.

Sample	H30S 3 M NaCl	H30S 3 M KCl	H50S 3 M NaCl
**Resilience <*****k*****> [N/m]**	0.016 ± 0.001	0.018 ± 0.002	0.034 ± 0.004
**MSD <*****u***^**2**^**> at 37 °C [Å**^**2**^]	17.9 ± 0.9	16.3 ± 0.8	12.1 ± 0.6

## References

[b1] Nissen P., Hansen J., Ban N., Moore P. B. & Steitz T. A. The structural basis of ribosome activity in peptide bond synthesis. Science 289, 920–930 (2000). 10937990 10.1126/science.289.5481.920

[b2] Krupkin M. . A vestige of a prebiotic bonding machine is functioning within the contemporary ribosome. *Trans R Soc Lond B Biol Sci* Sep 19 e-pub (2011). 10.1098/rstb.2011.0146 PMC3158926 21930590

[b3] Bashan A. & Yonath A. The linkage between ribosomal crystallography, metal ions, heteropolytungstates and functional flexibility. Journal of molecular structure 890, 289–294, doi: 10.1016/j.molstruc.2008.03.043 (2008). 19915655 PMC2757297

[b4] Yonath A. Large facilities and the evolving ribosome, the cellular machine for genetic-code translation. Journal of the Royal Society, Interface/the Royal Society 6 Suppl 5, S575–585, doi: 10.1098/rsif.2009.0167.focus (2009). PMC2843976 19656820

[b5] Zimmerman E. & Yonath A. Biological implications of the ribosome’s stunning stereochemistry. Chembiochem: a European journal of chemical biology 10, 63–72, doi: 10.1002/cbic.200800554 (2009). 19089882

[b6] Fischer N., Konevega A. L., Wintermeyer W., Rodnina M. V. & Stark H. Ribosome dynamics and tRNA movement by time-resolved electron cryomicroscopy. Nature 466, 329–333, doi: 10.1038/nature09206 (2010). 20631791

[b7] Frank J. & Agrawal R. K. A ratchet-like inter-subunit reorganization of the ribosome during translocation. Nature 406, 318–322, doi: 10.1038/35018597 (2000). 10917535

[b8] Schuwirth B. S. . Structures of the bacterial ribosome at 3.5 A resolution. Science 310, 827–834, doi: 10.1126/science.1117230 (2005). 16272117

[b9] Noeske J. . High-resolution structure of the Escherichia coli ribosome. Nature structural & molecular biology 22, 336–341, doi: 10.1038/nsmb.2994 (2015). PMC4429131 25775265

[b10] Horan L. H. & Noller H. F. Intersubunit movement is required for ribosomal translocation. Proceedings of the National Academy of Sciences of the United States of America 104, 4881–4885, doi: 10.1073/pnas.0700762104 (2007). 17360328 PMC1829233

[b11] Yonath A. The search and its outcome: High-Resolution Structures of Ribosomal Particles from Mesophilic, Thermophilic, and Halophilic Bacteria at Various Functional States. Annu. Rev. Biophys. Biomol. Struct. 31, 257–273 (2002). 11988470 10.1146/annurev.biophys.31.082901.134439

[b12] Sanbonmatsu K. Y. Computational studies of molecular machines: the ribosome. Current opinion in structural biology 22, 168–174, doi: 10.1016/j.sbi.2012.01.008 (2012). 22336622 PMC3675280

[b13] Chacon P., Tama F. & Wriggers W. Mega-Dalton biomolecular motion captured from electron microscopy reconstructions. Journal of molecular biology 326, 485–492 (2003). 12559916 10.1016/s0022-2836(02)01426-2

[b14] Wang Y., Rader A. J., Bahar I. & Jernigan R. L. Global ribosome motions revealed with elastic network model. Journal of structural biology 147, 302–314, doi: 10.1016/j.jsb.2004.01.005 (2004). 15450299

[b15] Trylska J., Tozzini V. & McCammon J. A. Exploring global motions and correlations in the ribosome. Biophys J 89, 1455–1463, doi: 10.1529/biophysj.104.058495 (2005). 15951386 PMC1366652

[b16] Zhang Z., Sanbonmatsu K. Y. & Voth G. A. Key intermolecular interactions in the E. coli 70S ribosome revealed by coarse-grained analysis. Journal of the American Chemical Society 133, 16828–16838, doi: 10.1021/ja2028487 (2011). 21910449 PMC3641354

[b17] Zimmermann M. T., Jia K. & Jernigan R. L. Ribosome Mechanics Informs about Mechanism. Journal of molecular biology, doi: 10.1016/j.jmb.2015.12.003 (2015). PMC4789072 26687034

[b18] Zaccai G. The ecology of protein dynamics. Current Physical Chemistry, Special Issue on Quantum Nanobiology and Biophysical Chemistry, Jalkanen K. J. Ed. 3, 9–16 (2013).

[b19] Brooks C. L., Karplus M. & Pettitt B. M. Proteins; a theoretical perspective of dynamics, structure and thermodynamics. Adv Chem Phys 71, 74–95 (1988).

[b20] Gabel F. . Protein dynamics studied by neutron scattering. Quarterly reviews of biophysics 35, 327–367 (2002). 12621860 10.1017/s0033583502003840

[b21] Hu X. . The dynamics of single protein molecules is non-equilibrium and self-similar over thirteen decades in time. Nature Physics 12, 171–174, doi: 10.1038/nphys3553 (2016).

[b22] Tehei M. . Adaptation to extreme environments: macromolecular dynamics in bacteria compared *in vivo* by neutron scattering. EMBO Rep 5, 66–70, doi: 10.1038/sj.embor.7400049 (2004). 14710189 PMC1298960

[b23] Schiro G. . Translational diffusion of hydration water correlates with functional motions in folded and intrinsically disordered proteins. Nature communications 6, 6490, doi: 10.1038/ncomms7490 (2015). PMC4382692 25774711

[b24] Mikl C. . Softness of atherogenic lipoproteins: a comparison of very low density lipoprotein (VLDL) and low density lipoprotein (LDL) using elastic incoherent neutron scattering (EINS). Journal of the American Chemical Society 133, 13213–13215, doi: 10.1021/ja203679g (2011). 21790144 PMC3173844

[b25] Peters J., Giudici-Orticoni M. T., Zaccai G. & Guiral M. Dynamics measured by neutron scattering correlates with the organization of bioenergetics complexes in natural membranes from hyperthermophile and mesophile bacteria. The European physical journal. E, Soft matter 36, 78, doi: 10.1140/epje/i2013-13078-y (2013). 23880731

[b26] Marty V. . Neutron scattering: a tool to detect *in vivo* thermal stress effects at the molecular dynamics level in micro-organisms. Journal of the Royal Society, Interface/the Royal Society 10, 20130003, doi: 10.1098/rsif.2013.0003 (2013). PMC3627083 23446053

[b27] Natali F., Gerelli Y., Stelletta C. & Peters J. Anomalous proton dynamics of water molecules in neural tissue as seen by quasi-elastic neutron scattering. Impact on medical imaging techniques. AIP Conf. Proc. 1518, 551, doi: 10.1063/1.4794632 (2013).

[b28] Harms J. . High resolution structure of the large ribosomal subunit from a mesophilic eubacterium. Cell 107, 679–688 (2001). 11733066 10.1016/s0092-8674(01)00546-3

[b29] Bonnete F., Madern D. & Zaccai G. Stability against denaturation mechanisms in halophilic malate dehydrogenase “adapt” to solvent conditions. Journal of molecular biology 244, 436–447, doi: 10.1006/jmbi.1994.1741 (1994). 7990132

[b30] Zaccai G. Hydration shells with a pinch of salt. Biopolymers 99, 233–238, doi: 10.1002/bip.22154 (2013). 23348670

[b31] Fitter J., Lechner R. E., Buldt G. & Dencher N. A. Internal molecular motions of bacteriorhodopsin: hydration-induced flexibility studied by quasielastic incoherent neutron scattering using oriented purple membranes. Proceedings of the National Academy of Sciences of the United States of America 93, 7600–7605 (1996). 8755521 10.1073/pnas.93.15.7600 PMC38792

[b32] Bellissent-Funel M. C. Hydration in protein dynamics and function. Joumal of Molecular Liquids 84, 39–52 (2000).

[b33] Ginzburg M., Sachs L. & Ginzburg B. Z. Ion Metabolism in a Halobacterium. I. Influence of age of culture on intracellular concentrations. Journal of General Physiology 55, 187–207 (1970). 5413077 10.1085/jgp.55.2.187 PMC2202994

[b34] Tehei M. . Neutron scattering reveals extremely slow cell water in a Dead Sea organism. Proceedings of the National Academy of Sciences of the United States of America 104, 766–771, doi: 10.1073/pnas.0601639104 (2007). 17215355 PMC1783388

[b35] Tehei M., Madern D., Pfister C. & Zaccai G. Fast dynamics of halophilic malate dehydrogenase and BSA measured by neutron scattering under various solvent conditions influencing protein stability. Proceedings of the National Academy of Sciences of the United States of America 98, 14356–14361, doi: 10.1073/pnas.251537298 (2001). 11734642 PMC64686

[b36] Li Z. Q. . Structure of phenylalanine-accepting transfer ribonucleic acid and of its environment in aqueous solvents with different salts. Biochemistry 22, 4380–4388 (1983). 6354255 10.1021/bi00288a006

[b37] Schober H. An introduction to the theory of nuclear neutron scattering in condensed matter. Journal of Neutron Research 17, 109–357 (2014).

[b38] Wood K., Lehnert U., Kessler B., Zaccai G. & Oesterhelt D. Hydration dependence of active core fluctuations in bacteriorhodopsin. Biophys J 95, 194–202, doi: 10.1529/biophysj.107.120386 (2008). 18339747 PMC2426655

[b39] Gallat F. X. . Dynamical coupling of intrinsically disordered proteins and their hydration water: comparison with folded soluble and membrane proteins. Biophys J 103, 129–136, doi: 10.1016/j.bpj.2012.05.027 (2012). 22828339 PMC3388209

[b40] Herschlag D., Allred B. E. & Gowrishankar S. From static to dynamic: the need for structural ensembles and a predictive model of RNA folding and function. Current opinion in structural biology 30, 125–133, doi: 10.1016/j.sbi.2015.02.006 (2015). 25744941 PMC4416989

[b41] Zaccai G. & Xian S. Y. Structure of phenylalanine-accepting transfer ribonucleic acid and of its environment in aqueous solvents with different salts. Biochemistry 27, 1316–1320 (1988). 3284581 10.1021/bi00404a034

[b42] Furtig B., Buck J., Richter C. & Schwalbe H. Functional dynamics of RNA ribozymes studied by NMR spectroscopy. Methods in molecular biology 848, 185–199, doi: 10.1007/978-1-61779-545-9_12 (2012). 22315070

[b43] Buck J. . NMR spectroscopic characterization of the adenine-dependent hairpin ribozyme. Chembiochem: a European journal of chemical biology 10, 2100–2110, doi: 10.1002/cbic.200900196 (2009). 19623596

[b44] Stadler A. M. . Thermal fluctuations of haemoglobin from different species: adaptation to temperature via conformational dynamics. Journal of the Royal Society, Interface/the Royal Society 9, 2845–2855, doi: 10.1098/rsif.2012.0364 (2012). PMC3479923 22696485

[b45] Caliskan G. . Dynamic transition in tRNA is solvent induced. Journal of the American Chemical Society 128, 32–33, doi: 10.1021/ja056444i (2006). 16390107

[b46] Réat V. . Dynamics of different functional parts of bacteriorhodopsin: H-2H labeling and neutron scattering. Proceedings of the National Academy of Sciences of the United States of America 95, 4970–4975 (1998). 9560212 10.1073/pnas.95.9.4970 PMC20197

[b47] Langer J. A., Engelman D. M. & Moore P. B. Neutron-scattering studies of the ribosome of Escherichia coli: a provisional map of the locations of proteins S3, S4, S5, S7, S8 and S9 in the 30 S subunit. Journal of molecular biology 119, 463–485 (1978). 347087 10.1016/0022-2836(78)90197-3

[b48] Ollivier J., Plazanet M., Schober H. & Cook J. C. First results with the upgraded IN5 disk chopper cold time-of-flight spectrometer. Physica B: Condensed Matter 350, 173–177 (2004).

[b49] Frick B. & Gonzalez M. Five years operation of the second generation backscattering spectrometer IN16—a retrospective, recent developments and plans. Physica B: Condensed Matter 301, 8–19 (2001).

[b50] Richard D., Ferrand M. & Kearley G. J. Analysis and visualisation of neutron-scattering data. J. Neutron Res. 4, 33–39, doi: 10.1080/10238169608200065 (1996).

[b51] Smith J. C. Protein dynamics: comparison of simulations with inelastic neutron scattering experiments. Quarterly reviews of biophysics 24, 227–291 (1991). 1749823 10.1017/s0033583500003723

[b52] Rahman A., Singwi K. S. & Sjölander A. Theory of Slow Neutron Scattering by Liquids. I. Phys. Rev. 126, 986–996 (1962).

[b53] Zaccai G. Neutron scattering perspectives for protein dynamics. J. Non-Cryst. Solids 357, 615–621 (2011).

[b54] Magazu S., Migliardo F. & Benedetto A. Mean square displacements from elastic incoherent neutron scattering evaluated by spectrometers working with different energy resolution on dry and hydrated (H2O and D2O) lysozyme. The journal of physical chemistry. B 114, 9268–9274, doi: 10.1021/jp102436y (2010). 20575549

[b55] Vural D., Hong L., Smith J. C. & Glyde H. R. Motional displacements in proteins: The origin of wave-vector-dependent values. Physical Review E 91, doi: 10.1103/PhysRevE.91.052705 (2015). 26066197

[b56] Réat V., Zaccai G., Ferrand M. & Pfister C. In Biological Macromolecular Dynamics (eds Cusack S. .) 117–122 (Adenine Press, 1997).

[b57] Wood K. . Dynamical heterogeneity of specific amino acids in bacteriorhodopsin. Journal of molecular biology 380, 581–591, doi: S0022-2836(08)00564-0 [pii]10.1016/j.jmb.2008.04.077 (2008). 18565346 10.1016/j.jmb.2008.04.077

[b58] Zaccai G. How soft is a protein? A protein dynamics force constant measured by neutron scattering. Science 288, 1604–1607 (2000). 10834833 10.1126/science.288.5471.1604

