# Supplementary material for: The fluctuating ribosome: thermal molecular dynamics characterized by neutron scattering
Source: Sci Rep. 2016 Nov 16;6:37138. doi: 10.1038/srep37138 (PMC5111069; doi:10.1038/srep37138)
Supplement: Supplementary Information [file srep37138-s1.pdf]

The fluctuating ribosome: thermal molecular dynamics characterized by neutron scattering

Giuseppe Zaccai[1, 2 *], Francesca Natali[1, 3], Judith Peters[1, 4], Martina Rihova[5, 6], Ella Zimmerman[8], J. Ollivier[1], J. Combet[1,7], Marie-Christine Maurel[5], Anat Bashan[8], Ada Yonath[8]

Affiliations:

1) Institut Laue Langevin, F-38042 Grenoble, France ; 2) Institut de Biologie Structurale (IBS), Univ. Grenoble Alpes, CEA, CNRS, 38044 Grenoble, France; 3) CNR-IOM, OGG, F-38042 Grenoble, France ; 4) Univ. Grenoble Alpes, LiPhy, F-38044 Grenoble, France ; 5) Institut de Systématique, Evolution, Biodiversité, ISYEB - UMR 7205- CNRS, MNHN, UPMC, EPHE UPMC, Sorbonne Universités, 57 rue Cuvier, CP 50, 75005 Paris, France ; 6) Institute of Physics, Charles University, Faculty of Mathematics and Physics, CZ-121 16 Prague, Czech Republic ; 7) Institut Charles Sadron, CNRS-UdS, 67034 Strasbourg Cedex 2, France ; 8) Weizmann Institute, Department of Structural Biology, 76100 Rehovot, Israel

*_**Corresponding author:** zaccai@ill.fr

Supplementary information

A diffusive motion produces QENS that can be described mathematically by a sum over Lorentzian functions, whose widths plotted as a function of Q^2 inform on motion parameters.

The dynamic structure factor for QENS is generally written

$$S(Q,\omega) = A_0(Q) \times \delta(\omega) + \left(1 - A_0(Q)\right) \times L(Q,\omega), \tag{4}$$

where ω is the energy transfer in units of $\hbar$, $A_0(Q)$ is the elastic incoherent structure factor (EISF), and $L(Q,\omega)$ is a Lorentzian function given by

$$L(Q,\omega) = \frac{1}{\pi} \frac{\Gamma(Q)}{\omega^2 + \Gamma(Q)^2}, \tag{5}$$

where $\Gamma(Q)$ is the half-width at half-maximum (HWHM).

A combination of three Lorentzians was here required to optimize the fits as reported in Figure S1.

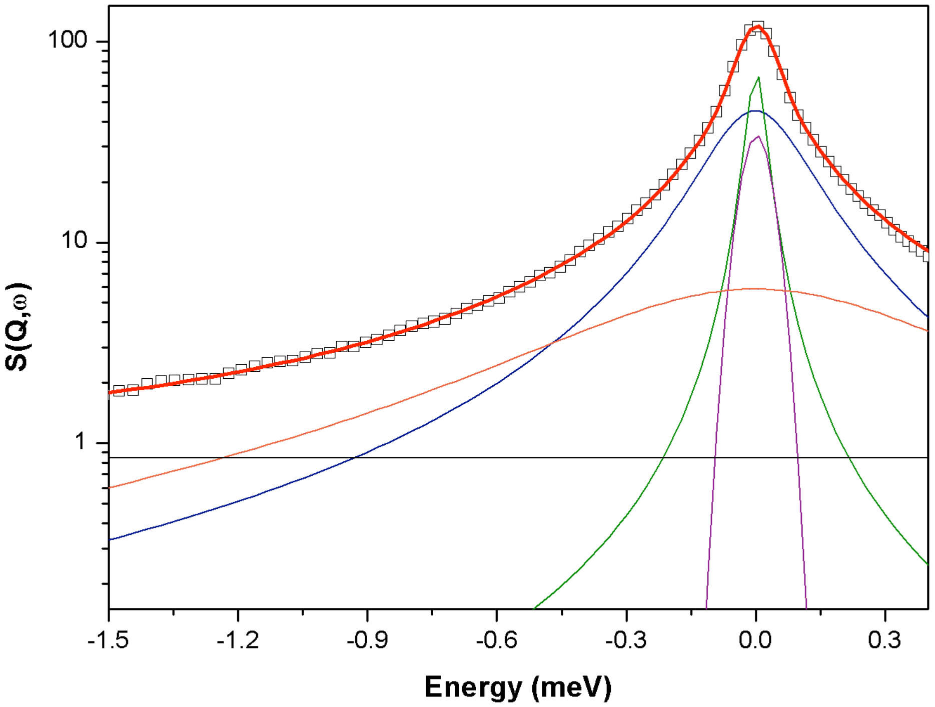

Figure S1. Fitting of the experimental spectrum (Q= 1.2 Å$^{-1,}$ black symbols) of H30S in NaCl by three Lorentzians and one delta function: L_1 (solid line, navy) and L_2 (solid line, green) refer to free and restricted water contributions, while L_3 (solid line, orange) represents a Q dependent broad background whose origin is still unclear. An additional background is considered (solid line, black). The best fit is indicated by a solid red line.

In the process of extracting information on the nature of the motions, the Q dependence of FWHM of the Lorentzians (Figure S2) were best fitted by a model in which, on average, H atoms diffuse with a coefficient D (cm^2/s) between sites with a residence time τ (s) confined inside a sphere of radius r (Å).

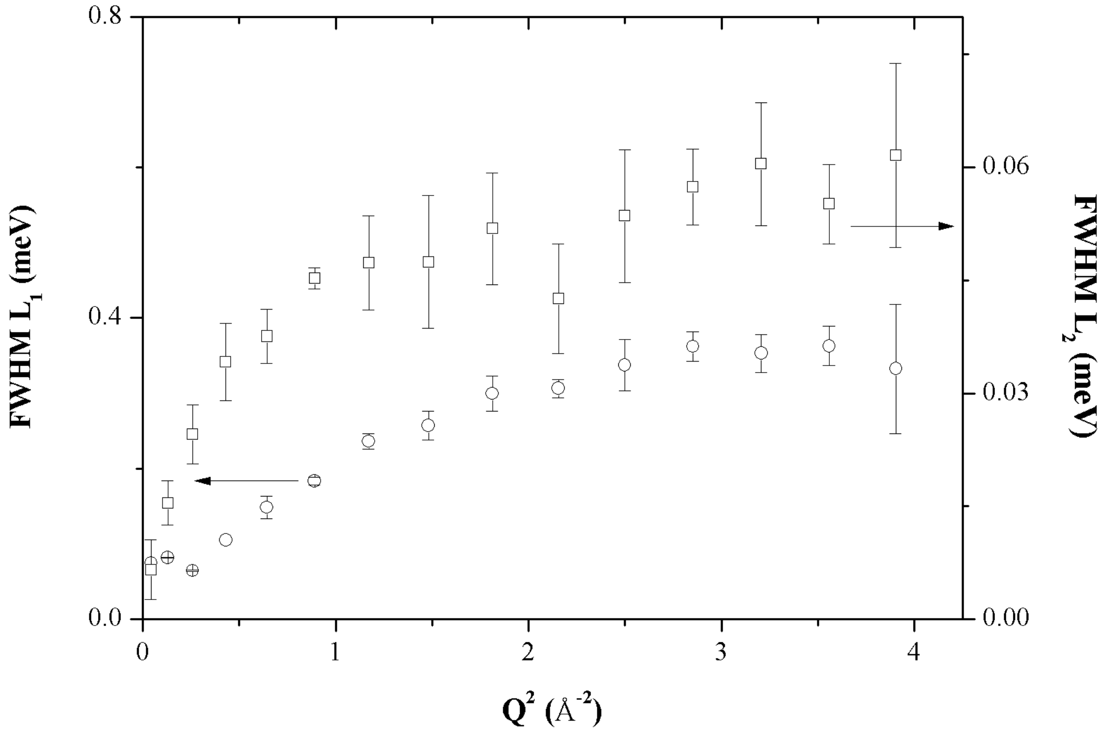

Figure S2: FWHM of the two main Lorentzian curves (L_1 and L_2) needed to fit QENS data of H30S in NaCl at 298 K.

Indeed, in the model developed by Singwi and Sjölander [1] for diffusive motions in water, hydrogen atoms jump between sites, separated by a length *l*, with a mean residence time τ. When the oscillatory period is much longer than τ the width of the quasi-elastic peak is given by:

$$\Gamma(Q) = \frac{1}{\tau}\left[1 - \frac{e^{-2W}}{1+Q^2 D\tau}\right], \qquad (6)$$

where $2W$ is a Debye-Waller term and D is a diffusion constant. When the Debye-Waller term is considered small, Γ is written

$$\Gamma(Q) = \frac{DQ^2}{1 + DQ^2\tau}, \qquad (7)$$

where l is related to D through $l^2 = 2D\tau$ [2].

For $Q^2 D\tau \gg 1$, the HWHM approaches its asymptotic value

$$\Gamma_\infty = \frac{1}{\tau}. \quad (8)$$

And for $Q^2 D\tau \ll 1$, the Singwi and Sjölander model reduces to continuous diffusion, for which $\Gamma = DQ^2$.

When the HWHM does not go to zero at $Q \rightarrow 0$ but tends to a constant value Γ_0, it is an indication of dynamics in a confined space. In the model of Volino and Dianoux [3], diffusion is confined to an impermeable sphere of radius r, where:

$$\Gamma_0 = 4.33\, D/r^2 \quad (9)$$

The corresponding fitting parameters for Lorentzians 1 and 2 are reported in Table S1.

Table S1. Fitting parameters: residence times, diffusion coefficients and sphere radii estimated through equations 7 to 9.

**Sample**	**H30S NaCl**		**H50S NaCl**	
	L_1	L_2	L_1	L_2
**τ [ps]**	17 ± 1	136 ± 9	18 ± 1	144 ± 9
**D [cm^2/s] (*10^{-5})**	2.8 ± 0.3	1.2 ± 0.2	3.0 ± 0.0.3	1.5 ± 0.3
**r [Å]**	4.8 ± 0.1		4.97 ± 0.06	

The values from the two subunits agree within errors, indicating that, on the ~10 ps timescale, the motion types are very similar for H30S and H50S. The two main Lorentzians ($L_1$and L_2) represent a fast confined motion and a slower not confined (on this timescale) diffusion, respectively, with parameters corresponding to free and hydration water and side-chain fast vibrations [4 5].

The Elastic Incoherent Structure Factor (EISF) shown in Figure S3 for H30S and H50S at 298K (25ºC) provides important information on the geometry of motion.

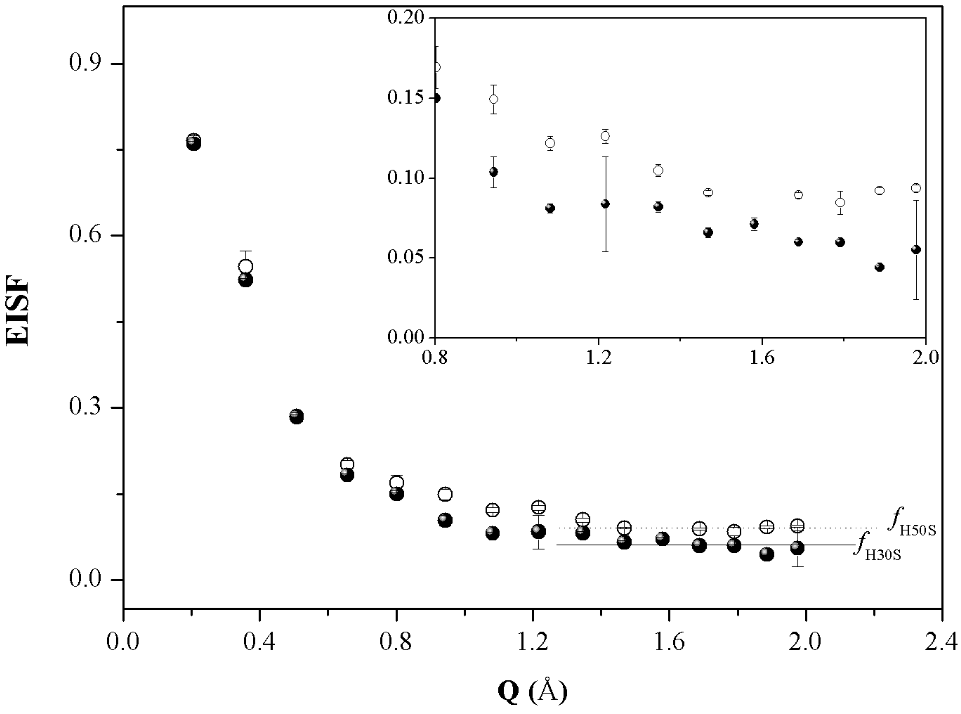

Figure S3. EISF of H30S and H50S at 298K (25ºC)

The two curves start to diverge outside the errors for Q-values above 0.7 $Å^{-1}$. The EISF tends to a plateau f at the higher Q-values where f is interpreted as the proportion of particles seen as 'immobile' within the instrumental resolution. The values of f estimated for the two

samples are f_{H30S} = 6.3 ± 0.5% and f_{H50S} = 9.3 ± 0.3%, indicating the larger proportion of atoms in H50S compared to H30S that are from stiffer regions whose motions are too slow to be resolved on the ~10 ps time scale.

Thus, the neutron scattering results on intra-molecular forces, diffusive fluctuation time scales and confinement geometries of motions in the halophilic H30S and H50S ribosomal subunits, given in Table SI-1, can be summarized as follows:

- On the ~10 ps timescale, which samples water diffusion and fast thermal vibrations, H30S and H50S mean dynamics are similar in terms of diffusion coefficient, residence time and confinement geometry of motion. H50S, however, is on average slower than H30S, displaying a slightly but significantly higher proportion than in H30S of domains in which atoms appear to be immobile on this time scale.

References

1. **Singwi, K. S. & Sjölander, A. (1960). Diffusive motions in water and cold neutron scattering. *Phys. Rev.* 119, 863-871.**
2. **Bee, M. (1988). *Quasielastic neutron scattering*, Adam Hilger, Bristol.**
3. **Volino, F. & Dianoux, A. J. (1980). Neutron incoherent scattering law for diffusion in a potential of spherical symmetry: general formalism and application to diffusion inside a sphere. *Mol. Phys.* 41, 271-279.**
4. **Bellissent-Funel, M. C. (2000). Hydration in protein dynamics and function. *Joumal of Molecular Liquids* 84, 39-52.**
5. **Fitter, J., Lechner, R. E., Buldt, G. & Dencher, N. A. (1996). Internal molecular motions of bacteriorhodopsin: hydration-induced flexibility studied by quasielastic incoherent neutron scattering using oriented purple membranes. *Proc Natl Acad Sci U S A* 93, 7600-5.**
